# Successful resection of a huge hepatocellular carcinoma during pregnancy: case report and review of the literature

**DOI:** 10.1186/s43046-025-00285-z

**Published:** 2025-06-16

**Authors:** Qihui Hu, Jiaxing Li, Jixing Wang, Cong Chen, Rui Tao

**Affiliations:** Bishan Hospital of Chongqing, Bishan Hospital of Chongqing Medical University, Chongqing, China

**Keywords:** Hepatocellular carcinoma, Hepatectomy, Pregnancy, Psychological support, Case report

## Abstract

**Background:**

Hepatocellular carcinoma during pregnancy is rare and poses significant potential risks to both the pregnant individual and the fetus. Here, we report a case of hepatocellular carcinoma during pregnancy. The 28-week gestational is a critical point of fetal maturation. A literature review revealed no similar case with survival exceeding 2 years, following resection of a large hepatocellular carcinoma diagnosed in late-stage pregnancy. This article may contribute to future research aimed at extending the survival time of patients with hepatocellular carcinoma diagnosed in late pregnancy.

**Case presentation:**

A 33-year-old pregnant woman was diagnosed with hepatocellular carcinoma at 34 weeks of pregnancy. A cesarean section was performed at 34 weeks of pregnancy. Under general anesthesia, a right lobectomy of the liver was conducted after 15 days. The patient received continuous support from the clinical psychology team throughout the entire perioperative period. The postoperative recovery was smooth, and the patient was discharged without any significant complications. Approximately 2 years post-surgery, follow-up indicated that the patient remained alive and in good health.

**Conclusions:**

The physiological changes associated with pregnancy can promote rapid tumor growth, leading to poor prognoses. Expert decision-making should be guided by the growth and maturation status of the fetus in relation to hepatocellular carcinoma development. For patients in the late stage of pregnancy, timely termination of pregnancy and tumor resection surgery, along with obtaining assistance from the clinical psychology team during the perioperative period, followed by post-discharge treatment with a combination of Sintilimab and Lenvatinib, constitutes an effective strategy for prolonging patient survival.

## Background

Hepatocellular carcinoma (HCC) during pregnancy represents an exceedingly rare clinical scenario that poses significant challenges in both diagnosis and management. While HCC typically arises in the context of chronic liver pathologies such as hepatitis B or C viral infections, chronic alcohol abuse, and non-alcoholic fatty liver disease [1], the unique physiological environment of pregnancy introduces additional complexity. Gestational hormonal fluctuations and immune system adaptations may potentially accelerate hepatocarcinogenesis, though the precise mechanisms remain unclear. Alpha-fetoprotein (AFP), a crucial serological marker for primary hepatic malignancies, demonstrates elevated levels in 70–95% of HCC cases, serving as a valuable tool for early detection and therapeutic monitoring. However, its diagnostic utility during pregnancy is compromised by physiological AFP elevation, particularly in the second and third trimesters [2].

As the third most prevalent malignancy worldwide with generally poor outcomes [3], HCC carries an exceptionally grave prognosis when coinciding with pregnancy. Current evidence indicates median survival of 18 months in this cohort, with survival rates declining from 50% at 6–12 months to 13.6% by 3–4 years post-diagnosis [4]. Although established etiological factors including chronic hepatitis B or C, aflatoxin exposure, and cirrhosis are well-characterized [5], the specific pathogenic mechanisms underlying pregnancy-associated HCC remain elusive, potentially involving synergistic effects of gestational hormonal shifts and immunomodulation.

The present study details a rare case of HCC diagnosed at 34 weeks of gestation, a critical period where fetal viability permits therapeutic intervention. Hepatocellular carcinoma diagnosed in the third trimester (≥ 28 weeks of gestation) exhibits a particularly poor prognosis, with no reported cases surviving beyond 2 years post-diagnosis. Notably, 28 weeks gestation represents a critical milestone for fetal development, marking the transition into the stage of maturation where viability significantly improves. Our systematic review of post- 2010 literature reveals no documented cases surviving beyond 24 months following surgical resection for HCC diagnosed in late pregnancy, highlighting the unique challenges in clinical management. These findings underscore the necessity for multidisciplinary management protocols and provide a foundation for future research directions aimed at improving outcomes for this vulnerable patient population.

## Case presentation

### Clinical presentation

A 33-year-old primigravida (G1P0) at 34 weeks gestation presented with persistent right upper quadrant pain. She experienced persistent epigastric discomfort that was not related to dietary intake. Upon her initial admission, she was found to have hepatitis B but had not received any routine treatment for it. The patient reported no history of hypertension, diabetes mellitus, heart disease, surgical procedures, trauma, or other medical conditions. On the abdominal examination, a large mass was noted in the right upper quadrant.

### Diagnostic workup

**Fig. 1:**
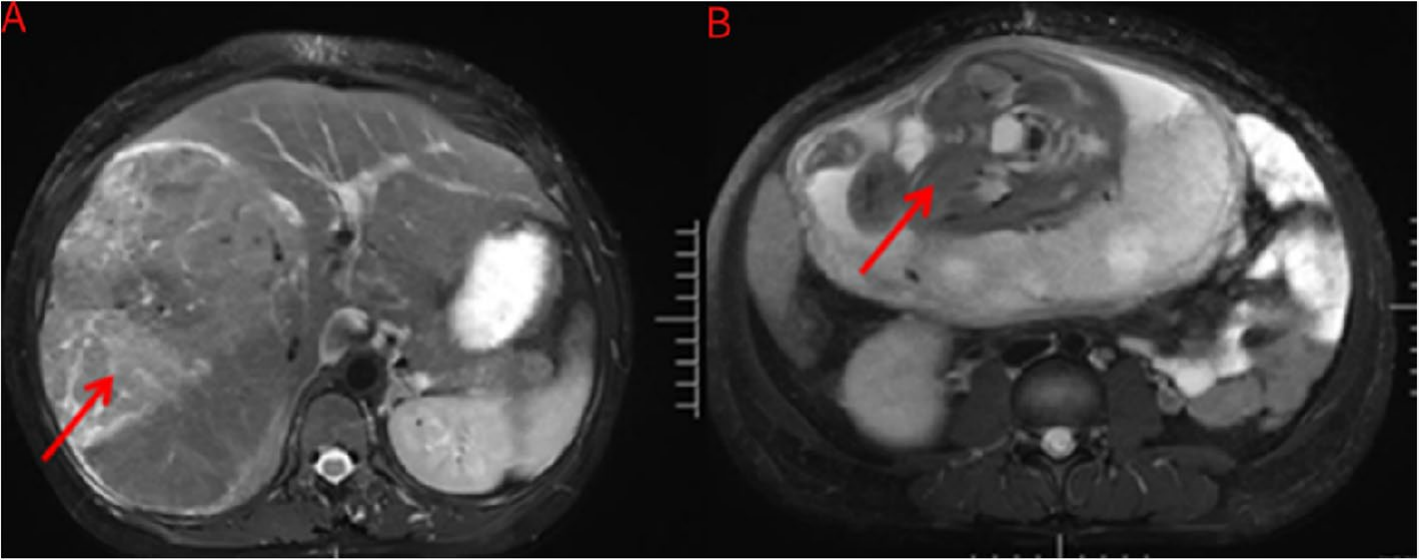
**A** Abdominal magnetic resonance imaging revealed a space occupying lesion of the right liver. **B** Fetal MRI was normal

Laboratory tests indicated anemia characterized by a hemoglobin level of 97 g/L, accompanied by elevated AFP measuring 139.67 ng/mL, and PIVKA-II 4926.39 mAU/mL. The HBV serological profile demonstrated positive results for HBsAg, HBeAg, and HBcAb. Quantitative HBV DNA testing was not performed. An abdominal ultrasound identified a slightly hyperechoic mass measuring 15.0 × 14.8 cm in the liver with unclear boundaries relative to surrounding tissues and an irregular shape. Abdominal magnetic resonance imaging (MRI) revealed a space-occupying lesion in the right lobe of the liver while fetal magnetic resonance imaging appeared normal as shown in Fig. 1.

**Fig. 2:**
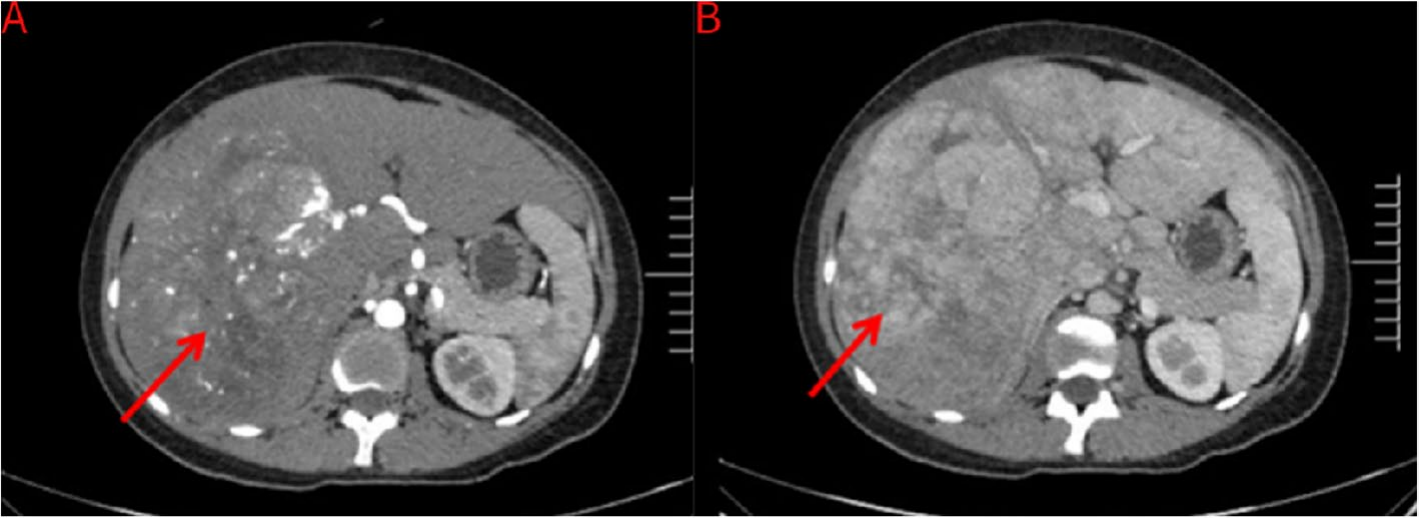
**A** Contrast enhanced CT revealed the mass had heterogeneous enhancement in the arterial phase. **B** Contrast-enhanced CT scan demonstrated no marked change in intensification in the venous phases

The possibility of HCC during pregnancy was considered. Given that the patient was still 34 weeks pregnant, delivery was deemed viable. However, surgery could not be postponed any longer due to the rapid growth of the tumor, which may have been associated with pregnancy. After thorough discussions within the multidisciplinary committee, which comprised experts from hepatobiliary surgery, obstetrics and gynecology, general surgery, and psychiatry, we obtained appropriate informed consent from the patient and their family. Subsequently, we decided to proceed with anesthesia in the subarachnoid space. The procedure went smoothly, resulting in the delivery of a healthy newborn. A contrast-enhanced abdominal CT performed 10 days later revealed a solid tumor measuring 20 × 18 cm that occupied the entire right abdomen and originated from the right lobe of the liver. The enhancement pattern observed on CT was characteristic of hepatocellular carcinoma as illustrated in Fig. 2. We utilized sonazoid-enhanced ultrasound to assist in diagnosis through parametric imaging. Additionally, we created a three-dimensional image that demonstrated the tumor's proximity to the inferior vena cava and right hepatic vein.

The patient’s HCC exhibited a maximum tumor diameter of 20 cm. Imaging and pathological evaluation revealed no invasion of the main branches of the portal vein or hepatic veins, along with absence of lymph node involvement and distant metastasis. Based on the American Joint Committee on Cancer (AJCC) staging system for HCC, this presentation is classified as Stage III.

**Fig. 3:**
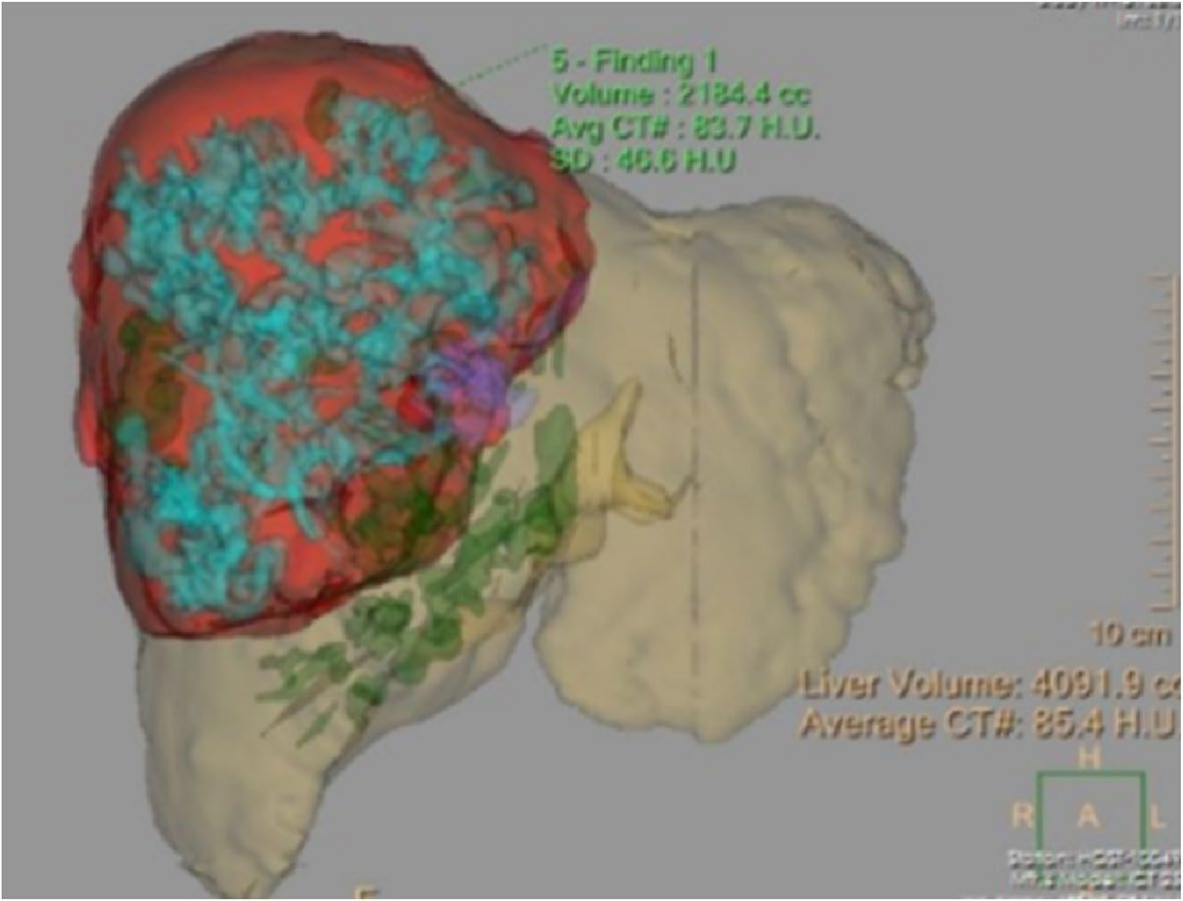
The three-dimensional image (VINCENT) showed the tumor adjacent to the inferior vena cava and right hepatic vein

We utilized sonazoid-enhanced ultrasound to assist in diagnosis through parametric imaging. Additionally, we created a three-dimensional image that demonstrated the tumor's proximity to the inferior vena cava and right hepatic vein (Fig. 3).

### Multidisciplinary management

**Fig. 4:**
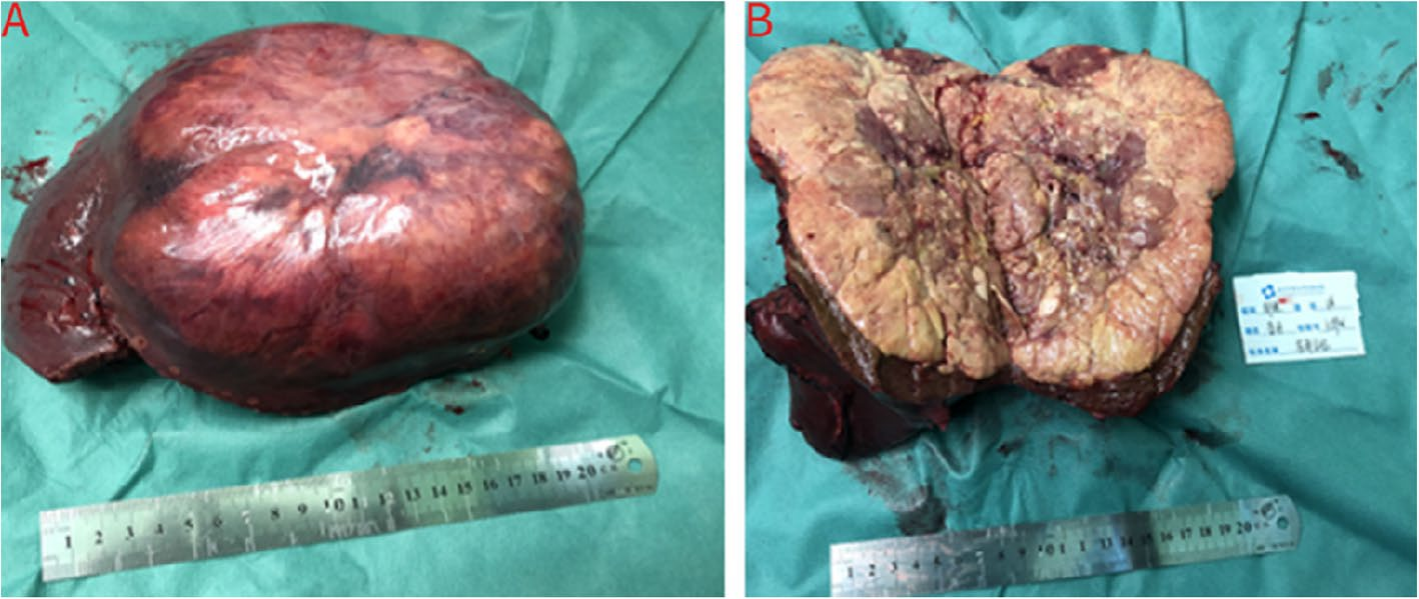
**A** Macroscopic aspect of the resected hepatocellular carcinoma. **B** The tumor exhibits an intact capsule in the cutting area, displaying a grey/white appearance

A cesarean section was performed at 34 weeks of gestation, followed by a right hepatectomy 15 days later. Macroscopic examination of the resected specimen revealed that the tumor measured approximately 225 mm in diameter and exhibited well-demarcated margins. While the tumor possessed a capsule, it lacked any septations as demonstrated in Fig. 4.

**Fig. 5:**
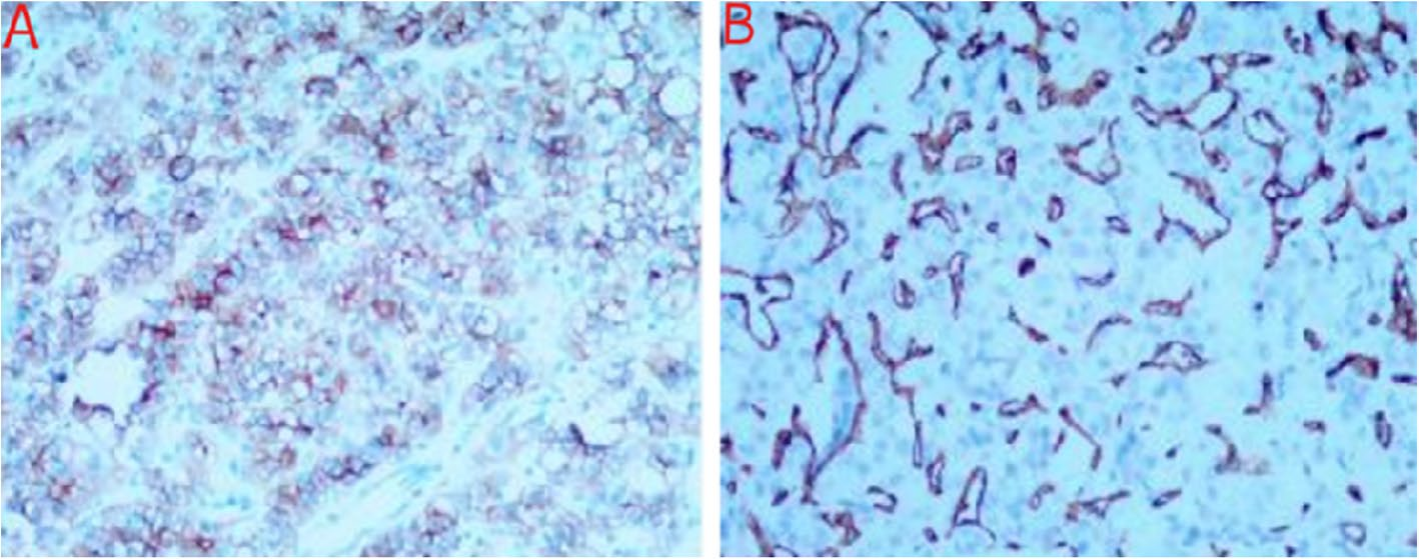
**A** The epithelial component of the tumor showed positive immunohistochemical staining for CK-L. **B** The stromal component revealed positive staining for CD34

Histopathological analysis confirmed the diagnosis of hepatocellular carcinoma. The epithelial component of the tumor showed positive immunohistochemical staining for CK-L. The stromal component revealed positive staining for CD34. The pathological results indicate a diagnosis of well-differentiated HCC as shown in Fig. 5. The patient demonstrated a satisfactory recovery and was discharged for 3 weeks post-surgery.

### Adjuvant therapy and follow-up

**Fig. 6:**
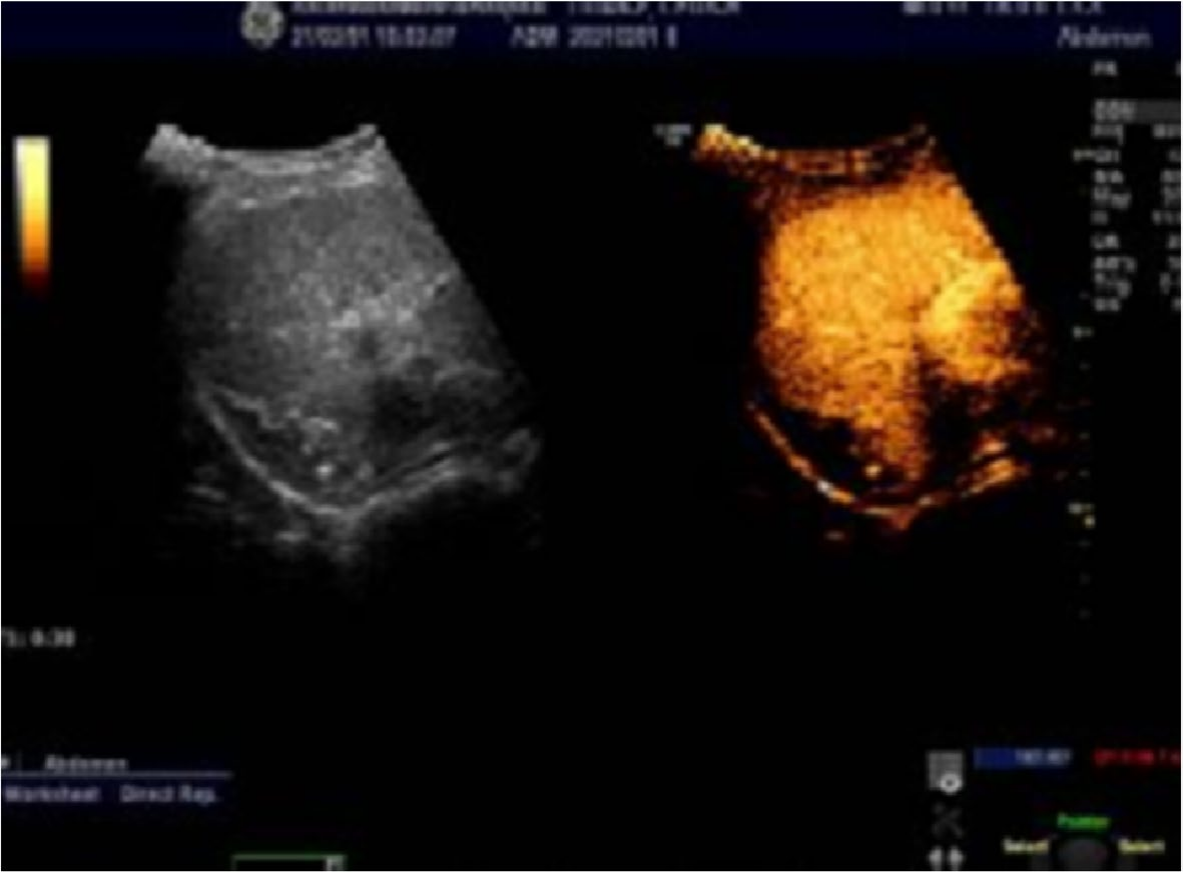
Sonazoid-enhanced US performed 2 months after surgery revealed no recurrence or metastasis

Postoperatively, the patient was administered Sintilimab, a programmed cell death protein- 1 (PD- 1) inhibitor, and Lenvatinib at a dose of 8 mg/day. Throughout the perioperative period, a clinical psychology team was engaged to implement multidimensional psychological support, aiming to stabilize emotional well-being and enhance treatment adherence. Interventions included: first, identifying treatment-related anxiety via the Hospital Anxiety and Depression Scale. Second, administering cognitive behavioral therapy (CBT) to address misconceptions about immunotherapy, implementing mindfulness-based stress reduction incorporating Sonazoid-enhanced ultrasound imaging for positive visualization, and developing family support system development. Third, conducting multidisciplinary consultations to differentiate psychosomatic symptoms and tailor strategies for managing drug side effects. These interventions significantly reduced anxiety scores and improved treatment compliance. Follow-up assessments indicated that the patient remains in stable condition, and sonazoid-enhanced ultrasound revealed no signs of recurrence or metastasis (Fig. 6).

## Discussion

The early diagnosis of HCC during pregnancy poses significant challenges due to its insidious onset. Implementing multiple auxiliary examinations for pregnant patients can enhance the detection rate of HCC. To mitigate potential adverse effects of radiographic imaging on the fetus, B-ultrasound and MRI are considered optimal modalities for preoperative diagnosis of hepatocellular carcinoma in this population. In this case report, the patient underwent an abdominal ultrasound, which revealed a large HCC. Subsequently, abdominal CT and MRI were performed. These imaging techniques proved valuable in determining the origin of the HCC, assessing the extent of tumor invasion, and facilitating differential diagnosis from various organ tumors.

Several studies have shown that pregnancy clearly has an adverse effect on the prognosis of HCC [6–8]. HCC becomes more aggressive during pregnancy, primarily due to two main etiologies: estrogen elevation and immune suppression during pregnancy. Accordingly, estrogen has been demonstrated to enhance hepatocyte mitosis, increase hypervascularity, and elevate free radical levels. Additionally, it may reactivate the hepatitis B virus and diminish humoral immunity. During pregnancy, large amounts of human chorionic gonadotropin, estrogen, and placental lactogen secreted from the placenta are believed to promote the growth and reproduction of cancer cells, thus aggravating the aggressiveness of the underlying HCC [9]. Moreover, gestational immune suppression may be an enabling factor for tumor progression [10]. Thus, the pregnancy was terminated as soon as possible for hepatocellular carcinoma patients.

We performed a systematic literature search spanning from database inception to 2024 across four biomedical databases (PubMed, MEDLINE, Embase, Cochrane Library). The search protocol utilized Boolean logic to combine MeSH terms and title keywords: (pregnancy (Title) AND “hepatocellular carcinoma” (MeSH Terms)) OR (“hepatocellular carcinoma” (Title) AND pregnancy (Title)). Our systematic review of literature since 2010 (summarized in Table 1 [11–26]) revealed two critical findings: First, no documented cases exist of late-pregnancy HCC patients surviving beyond 2 years post-surgical resection. Second, comparative analysis with historical case reports demonstrates that delayed diagnosis and subsequent surgical intervention in pregnancy-associated HCC correlate with significantly reduced survival durations. Notably, our current case exhibits distinct clinical characteristics when juxtaposed with previously reported cases.

**Table 1:** The reports of hepatocellular carcinoma during pregnancy

Case	Author	Age	Disease	HCC stage (AJCC)	Weeks of diagnosis	Survival after diagnosis
1	Cao MK [11]	29	HBsAg carrier	II	21	Unknown
2	Seaward PG [12]	17	Unknown	Unknown	32	Unknown
3	Hung CC [13]	30	Unknown	II	25	> 48 months
4	Norouzi [14]	41	N	II	22	< 1 months
5	Li [15]	27	HBsAg carrier	III	32	12 months < × < 24 months
6	Iijima T [16]	40	*N*	III	30	Unknown
7	Gerli [17]	30	*N*	III	30	6 months < × < 12 months
8	Russell P [18]	33	*N*	Unknown	30	Unknown
9	Qasrawi [19]	38	*N*	III	36	6 months
10	Francis [20]	36	Unknown	Unknown	23	2 months < × < 6 months
11	Monteiro de [21]	36	HBsAg carrier	III	33	< 1 months
12	McCarthy CM [22]	39	N	III	11	Unknown
13	Grubman [23]	37	Unknown	Unknown	29	6 months
14	Diakhate [24]	30	Unknown	Unknown	31	< 2 months
15	Maeda T [25]	36	HBsAg carrier	II	20	> 48 months
16	Marasciulo F [26]	35	HBsAg carrier	Unknown	30	< 6 months
17	Marasciulo F [26]	38	N	Unknown	32	< 6 months
18	Present case	33	HBsAg carrier	III	34	> 24 months

A staged surgical treatment protocol was performed based on the Barcelona Clinic Liver Cancer (BCLC) staging classification. The option of termination of pregnancy: fetus could not survive if the gestational week of less than 28 weeks; the fetus could survive if the gestational week of greater than or equal to 28 weeks, the patient underwent emergency cesarean section for huge HCC. In this case report, the cesarean section was performed at 34 weeks. Our case represents a unique clinical scenario in three aspects: First, the patient achieved the longest reported survival (2 years) following HCC resection during late pregnancy. Second, the combination therapy of Sintilimab and Lenvatinib post-surgery may have contributed to sustained remission, as evidenced by the absence of recurrence on imaging. Third, we introduced a mental health team during the entire surgery process—they used CBT to manage anxiety before surgery and provided emotional support after operations. This whole-person care method hasn’t been routinely used in previous cases like this.

Sintilimab, a PD- 1 inhibitor, functions by blocking the PD- 1/PD-L1 signaling axis to reactivate T cell-mediated antitumor immunity. Lenvatinib, a multitargeted tyrosine kinase inhibitor, suppresses tumor angiogenesis and proliferation through inhibition of VEGFR, FGFR, and PDGFR pathways. Preclinical studies demonstrate that lenvatinib enhances intratumoral CD8 + T cell infiltration while reducing immunosuppressive regulatory T cells, creating an immunologically favorable microenvironment that potentiates PD- 1 inhibitor efficacy. This synergistic mechanism translates to significant survival benefits in advanced HCC, with combination therapy demonstrating superior progression-free survival and overall survival compared to monotherapies [27]. The 2024 guidelines endorse targeted-immunotherapy combinations (PD- 1 inhibitors + TKIs) as a promising adjuvant strategy following surgical resection, particularly for high-risk HCC cases with vascular invasion or suboptimal tumor differentiation [28]. The support and assistance provided by the clinical psychology team during this time play a crucial role in the overall treatment and rehabilitation of patients. Preoperatively, patients may experience anxiety and depressive symptoms due to fears and concerns regarding the surgery. The clinical psychology team assesses the psychological state of these patients and offers appropriate interventions, such as CBT, to help alleviate preoperative anxiety and enhance their confidence and preparedness for the procedure. Postoperatively, patients might face psychological issues stemming from pain at the surgical site, uncertainties about recovery, or worries about disease prognosis. The clinical psychology team can assist by managing pain effectively while providing emotional support that allows for emotional expression, thereby facilitating psychological recovery in these individuals [29]. Our research findings suggest that the presence of well-differentiated tumor characteristics in patients, combined with proactive and appropriate treatment strategies, as well as the involvement of a clinical psychology team in the therapeutic process, significantly contributes to achieving favorable clinical outcomes. Consequently, this approach has led to a notable extension of survival time for patients during late-stage pregnancy compared to other patients.

Our study has limitations inherent to single-case reports. The absence of long-term follow-up beyond 2 years precludes definitive conclusions about cure rates. Additionally, the role of adjuvant Sintilimab-Lenvatinib therapy in pregnancy-associated HCC requires validation through larger cohorts, particularly given potential safety concerns in postpartum women. Finally, the impact of psychological interventions, while promising, warrants standardized assessment tools to quantify their contribution to survival.

## Conclusions

This case report underscores the rarity and complexity of HCC during pregnancy, a condition that presents significant challenges to both maternal and fetal health. The successful management of this case, resulting in the patient’s survival of more than 2 years postoperatively, underscores the critical importance of a multidisciplinary approach. This approach involves not only surgical intervention but also the timely termination of pregnancy and the use of novel combination therapies such as Sintilimab and Lenvatinib. The integration of a clinical psychology team throughout the perioperative period further demonstrates the value of addressing the patient’s emotional and psychological needs as part of the overall treatment strategy. This comprehensive management strategy, which includes psychological support, may have contributed significantly to the patient’s sustained remission and improved quality of life. Future research should focus on validating the effectiveness of such multidisciplinary approaches, including the role of adjuvant therapies and psychological interventions, in larger cohorts to better understand their impact on patient outcomes and survival rates.

## Data Availability

Data availability is not applicable to this case report as no new datasets were generated. All clinical findings and analyses are based on previously published studies cited in the references.
